# Integrating Artificial Intelligence into Breast Cancer Histopathology: Toward Improved Diagnosis and Prognosis

**DOI:** 10.3390/cancers18071184

**Published:** 2026-04-07

**Authors:** Gavino Faa, Eleonora Lai, Flaviana Cau, Ferdinando Coghe, Massimo Rugge, Jasjit S. Suri, Claudia Codipietro, Benedetta Congiu, Simona Graziano, Ekta Tiwari, Andrea Pretta, Pina Ziranu, Mario Scartozzi, Matteo Fraschini

**Affiliations:** 1 Department of Medical Sciences and Public Health, University of Cagliari, AOU Cagliari, 09124 Cagliari, Italy; gavinofaa@gmail.com (G.F.); flacau@tiscali.it (F.C.); 2 Department of Biology, College of Science and Technology, Temple University, Philadelphia, PA 19122, USA; 3 Medical Oncology Unit, University Hospital and University of Cagliari, 09042 Cagliari, Italy; claudiacodipietro96@gmail.com (C.C.); benedettacongiu95@gmail.com (B.C.); s.graziano@aoucagliari.it (S.G.); an.pretta@gmail.com (A.P.); pi.ziranu@gmail.com (P.Z.); marioscartozzi@unica.it (M.S.); 4 Clinical-Microbiological Laboratory, University Hospital of Cagliari, 09042 Cagliari, Italy; fcoghe@aoucagliari.it; 5 Department of Medicine–DIMED, General Anatomic Pathology and Cytopathology Unit, Università degli Studi di Padova, 35121 Padova, Italy; massimo.rugge@unipd.it; 6 Stroke Monitoring and Diagnostic Division, AtheroPoint LLC, Roseville, CA 95661, USA; jasjit.suri@atheropoint.com; 7 Department of Electrical and Computer Engineering, Idaho State University, Pocatello, ID 83209, USA; 8 Department of Innovation, Global Biomedical Technologies, Inc., Roseville, CA 95661, USA; ekta.tiwari.ai@gmail.com or; 9 Symbiosis Institute of Technology, Nagpur Campus, Symbiosis International (Deemed University), Pune 440008, India; 10 Department of Electrical and Electronic Engineering, Università degli Studi di Cagliari, 09123 Cagliari, Italy; fraschin@unica.it

**Keywords:** breast cancer, artificial intelligence, digital pathology, whole slide imaging, deep learning, histopathology, diagnosis and prognosis, computational pathology

## Abstract

Histopathological evaluation of tissue sections remains the gold standard for the diagnosis, classification, and grading of breast cancer (BC). The widespread adoption of whole-slide imaging (WSI) has enabled the digitization of histological slides and facilitated the development of artificial intelligence (AI) approaches for computational pathology. In recent years, machine learning and deep learning (DL) algorithms have been increasingly investigated for the analysis of hematoxylin and eosin (H&E)-stained images, with potential applications in tumor detection, histological classification, prognostic stratification, and prediction of treatment response. This narrative review summarizes recent developments in AI-driven models applied to BC histopathology and discusses their potential role in supporting diagnostic and prognostic assessment. Several studies have demonstrated the promising performance of DL algorithms in tasks such as the detection of lymph node metastases, assessment of residual tumor after neoadjuvant therapy, and prediction of clinical outcomes from histopathological images. Emerging research has also explored the possibility of inferring molecular and biomarker information from histology images, although these approaches currently identify statistical associations rather than direct molecular measurements. Despite the rapid expansion of this research field, significant barriers remain before routine clinical implementation can be achieved. Key challenges include dataset bias, variability in staining and image acquisition, limited external validation across institutions, and the need for transparent and reproducible model development. In addition, the translation of AI-based systems into clinical practice requires compliance with regulatory frameworks governing software used for medical purposes, such as those established by the U.S. Food and Drug Administration. Overall, AI represents a promising research direction in computational pathology and may contribute to decision-support tools capable of assisting pathologists in the analysis of digital slides. Continued efforts toward methodological rigor, large multicenter datasets, and prospective validation studies will be essential to determine the future role of AI in BC histopathology.

## Simple Summary

Breast cancer (BC) diagnosis and prognosis are traditionally based on the microscopic evaluation of hematoxylin and eosin (H&E)-stained tissue sections. The introduction of whole-slide imaging has enabled the digitization of histological slides and opened the possibility of applying artificial intelligence (AI) techniques to digital pathology. Recent studies have explored the use of deep learning algorithms to analyze histological images for tasks such as tumor detection, identification of lymph node metastases, and assessment of tumor characteristics relevant for prognosis. Some research has also investigated whether patterns in routine histology images may correlate with molecular biomarkers such as hormone receptor status or HER2 expression. However, these approaches currently identify statistical associations rather than replacing established laboratory tests. AI-based tools are therefore mainly being developed as decision support systems that may assist pathologists in the interpretation of digital slides. Despite promising research results, several challenges still limit the routine clinical implementation of AI in pathology. These include dataset bias, limited external validation across institutions, and the need to comply with regulatory frameworks governing medical software, such as those established by the U.S. Food and Drug Administration. Overall, AI represents an emerging research area in digital pathology and may contribute to improved analysis of BC histopathology in the future, supporting pathologists in diagnostic and prognostic evaluation.

## 1. Introduction

Histopathology based on microscopic examination of tissue sections, remains the gold standard for the diagnosis, classification and grading of breast cancer (BC) [1]. The evaluation of hematoxylin and eosin (H&E)-stained tissue sections enables the identification of tumor architecture, cellular morphology, and features relevant for tumor grading, staging and treatment, such as hormone receptor status [1,2].

The introduction of whole-slide imaging (WSI), a technology that allows the digital acquisition of histological slides at high resolution, has progressively transformed pathology workflows [3]. Digital pathology is transforming the traditional pipeline of pathology practice, based on the analysis of tissues under the microscope, into a computer vision workflow [4]. Indeed, it facilitates image sharing, archiving, and computational analysis, thereby enabling the application of artificial intelligence (AI) algorithms to histopathological images, presenting a novel, unique perspective in oncology [5]. In recent years, machine learning (ML) and deep learning (DL) models have been increasingly explored in computational pathology, including applications in BC histopathology, with the aim to make AI systems more transparent and explainable [6,7].

The new AI-driven models demonstrate the potential to predict molecular changes in cancer cells based on histology alone, including histological phenotypes related to different steps of carcinogenesis and microsatellite instability [8,9,10]. Given the dimensionality of WSI, their automatic segmentation into multiple smaller patches has been introduced in order to elevate precision, speed and reproducibility of histological cancer images [3].

**Figure 1:**
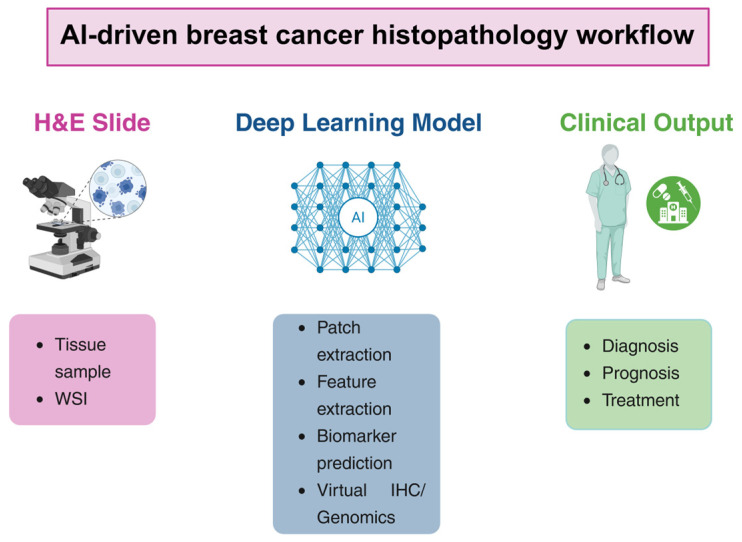
AI-driven breast cancer histopathology workflow. Abbreviations: AI, artificial intelligence; H&E, hematoxylin and eosin; IHC, immunohistochemistry; WSI, whole-slide imaging.

These methods aim to extract quantitative features from digital slides and to assist pathologists in tasks such as tumor detection, grading, biomarker prediction, and prognostic stratification. Convolutional neural networks (CNNs) and other DL architectures can analyze large image datasets and learn hierarchical feature representations directly from histological images. DL models transform high-dimensional inputs like histological images to numbers representing event times for survival analysis, class probabilities for classification or other images for segmentation via the intermediate step of translating inputs into representations. When the new DL model is trained, the network learns how to produce representations that capture the appropriate structure so that they can be converted to the desired output (Figure 1) [6].

However, despite the rapid growth of this research field, the translation of AI-based models into clinical practice remains limited. Many studies rely on retrospective datasets, lack external validation, or do not address methodological issues such as dataset bias, domain shift, and reproducibility. Furthermore, regulatory pathways and clinical integration strategies are still evolving, and pathologists are asking for global regulation in this field, in order to facilitate the employment of ML and DL models in clinical practice [11].

The aim of this narrative review is to critically summarize recent developments in the application of AI-driven models to BC histopathology, focusing on diagnostic and prognostic applications using H&E-stained slides. Particular attention is given to methodological challenges, dataset limitations, and the requirements for clinical translation of computational pathology tools.

## 2. The Performance of AI-Driven Models in the Diagnosis of BC

Early applications of computational pathology in BC focused on the automated detection of lymph node metastases in digitized histological slides [12,13,14]. Landmark challenges such as CAMELYON demonstrated that deep learning models could achieve high sensitivity in identifying metastatic deposits in lymph nodes when trained on large annotated datasets [12]. With the increasing adoption of WSI technology in pathology laboratories, multiple studies have investigated the use of AI models for tumor detection, tissue classification, and histological pattern recognition in BC specimens. These approaches typically rely on CNNs trained on either WSI or image patches extracted from WSI [3,15,16]. The imperative “Train longer, generalize better” has been proposed as a trick to obtain better and more generalizable results using large datasets when applying DL models to BC analysis, but it still requires validation [17,18]. In patch-based approaches, WSI are subdivided into smaller image tiles that can be processed by DL algorithms. Although this strategy facilitates model training, it introduces potential methodological challenges such as tile-level data leakage and loss of spatial context. More recent approaches attempt to address these limitations using weakly supervised learning or multiple-instance learning frameworks that operate at the slide level.

While several studies have reported promising diagnostic performance, it is important to emphasize that many of these models have been evaluated primarily on retrospective datasets and under controlled experimental conditions. External validation on independent multicenter cohorts remains relatively limited, which restricts the assessment of their generalizability across different scanners, staining protocols, and patient populations [19].

Table 1 shows representative studies applying AI methods to BC histopathology.

## 3. AI-Driven Models in the Neoadjuvant Setting of BC

Further studies compared WSI-based and patch-based sampling strategies for detecting BC cells following neoadjuvant therapy [19]. The effect of different types of image augmentation on classification tasks was also assessed. The initial studies on WSI of cancer were carried out on patch-based annotations, which are time-consuming and not feasible in routine clinical practice. To avoid the annotation burden, more recent studies proposed weak supervision methods and annotation-free approaches capable of training DL models to explore relationships inside cancer WSI [20,28]. Digital pathology was proposed for the assessment of residual BC cellularity following neoadjuvant chemotherapy [29]. Interestingly, AI-driven models have been shown to work well even on frozen sections, shortening times for digital pathology-based diagnosis and enhancing cancer classification [30].

AI-driven models have also been proposed for the prediction of the response to neoadjuvant chemotherapy based solely on the analysis of BC histopathological images [31].

## 4. AI-Driven Models and Prognosis in BC

Another relevant ability of DL systems is the prediction of overall survival (OS) in patients affected by multiple cancer types, including BC, using solely histopathological images of cancer biopsies [32]. Very recently, the development of a new DL framework, named ResoMergeNet, represented a revolution in BC diagnosis and prognostication [33]. This new DL model showed superior performance against state-of-the-art models, paving the way for precise BC diagnosis and prognosis and opening new frontiers in the field of histopathological image analysis. In the near future, by enhancing the model’s explainability, ResoMergeNet might be validated, enabling its introduction and integration into clinical workflows in pathology and oncology departments.

## 5. AI-Driven Models for Biomarker Prediction and Tumor Classification

An emerging research direction in computational pathology is the prediction of molecular biomarkers directly from histological images. Several studies have investigated whether DL models trained on H&E-stained slides can infer the status of clinically relevant biomarkers such as HER2 expression or hormone receptor (HR) status.

These approaches are sometimes described as “virtual immunohistochemistry (IHC)”. However, it is important to emphasize that such models currently identify statistical correlations between morphological patterns and molecular alterations rather than directly measuring protein expression or gene status. Consequently, AI-based biomarker prediction should not be interpreted as a replacement for established molecular or immunohistochemical assays without rigorous validation [34].

Some studies have reported promising performance in predicting HER2 status from histological images, suggesting that morphological features may correlate with underlying molecular characteristics [35,36,37]. Similar approaches have been explored for the prediction of estrogen receptor and progesterone receptor status [38].

A further improvement in the ability of AI-driven models to extract subtle features from the histopathology of BC alone came from studies showing the ability of DL models to give information regarding the prediction of gene expression and of the transcriptomic profile in BC cells from H&E-stained sections, opening the way for a new field of digital pathology: virtual genetics and transcriptomics of cancer [39,40,41,42].

An improvement in the approach to BC pathological classification by using DL models applied to histological images came from the proposal of a two-step approach: first, a ×4 image to locate the regions of interest (ROI), i.e., cancer cells, in the WSI and, second, ×40 images of ROI that should be utilized for the final image recognition task [43]. Interestingly, this process would closely resemble the approach used by pathologists in real-life clinical practice to analyze cancer histological images under the microscope.

In short, AI-driven models might represent a promising approach for detecting BC cells, better classifying BC histopathology images, improving cancer grading, refining prognostic classification, and predicting clinical benefit from adjuvant chemotherapy [14,44,45,46,47,48,49,50,51]. Nevertheless, the biological mechanisms underlying these correlations remain incompletely understood. Potential confounding factors such as tumor subtype, grade, and dataset composition may influence model performance. Therefore, further studies incorporating external validation, prospective evaluation, and orthogonal molecular testing are necessary before such approaches could be considered for clinical use.

## 6. Reducing Inter-Observer Variability in BC Histopathology: AI Applications of AI-Assisted Pathology and Inter-Observer Variability

Histopathological interpretation may be affected by inter-observer variability, particularly in tasks such as tumor grading or biomarker scoring. AI-based image analysis tools have been proposed as potential decision support systems that could assist pathologists in performing quantitative or repetitive tasks.

Several frameworks have been developed to facilitate the integration of deep learning algorithms into digital pathology workflows. For example, libraries designed for WSI image preprocessing and annotation management may help standardize data preparation steps and improve reproducibility in computational pathology studies. An important step in the validation process of WSI-based AI-driven systems is represented by the approval of the FDA of a system for routine pathology diagnostic purposes in the United States [52]. This approval favored the development of new AI-driven models for their introduction into the clinical workflow in pathology departments. The development of a new self-supervised DL model, named SISH (self-supervised image search for histology), represented a promising direction in the introduction of AI models in oncology [53]. The SISH algorithm provides an open-source package, requiring only slide-level annotations for training, and was proposed as a tool for pathologists in the diagnosis of cancer, including tumors of unknown primary [54]. In this article, the following key challenges in WSI search were identified: speed, accuracy, scalability, constant search speed, and strong performance on diverse datasets. One of the aims of this work was the proposal of a new AI-driven model able to find a solution for one of the most relevant problems in human histopathology: the removal of inter-observer variability in histopathological diagnosis [55].

In order to decrease interobserver variability among pathologists in different fields of human pathology, including cancer, a new DL method, named SliDL, has been developed to perform pre- and post-processing WSI [56,57]. SliDL is a Python library that simplifies many of the steps required to tackle the challenges posed by WSI technology. SliDl is unique in its support for annotation handling and for empowering pathologists to accelerate the application of DL systems in routine pathology practice within the clinical workflow [58].

However, it would be inaccurate to assume that AI systems eliminate diagnostic variability. Instead, AI introduces different sources of variability related to training data, algorithm design, and dataset bias. Consequently, AI tools should be viewed as assistive technologies that complement the expertise of pathologists rather than replace human interpretation.

## 7. Comprehensive Application of AI-Driven Models in BC

In recent years, advanced algorithms and CNNs have been augmenting pathologists’ diagnostic abilities, opening new frontiers in automated image analysis of cancer histopathological images. These advancements in digital pathology are unraveling the potential of AI for precision diagnosis and prognosis of BC [59]. WSI technology represents a paradigm shift in pathology departments, a fundamental step for allowing a wide array of digital tools, including ML and DL algorithms, to enter the field of cancer histopathology and clinical oncology [60]. WSI represents a potential opportunity for pathologists to guide the new AI-driven technology in cancer image analysis, improving the standardization of cancer diagnosis and enabling the extraction of subtle features from histology, thereby providing oncologists with relevant molecular and prognostic information.

**Figure 2:**
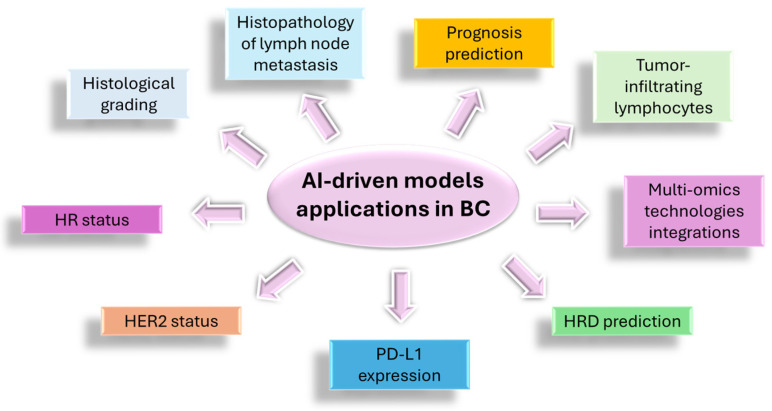
Applications of AI-driven models in breast cancer. Abbreviations: AI, artificial intelligence; BC, breast cancer; HR, hormone receptors; HER2, Human Epidermal Growth Factor 2; HRD, Homologous recombination deficiency; PD-L1, programmed death ligand-1.

Two recent reviews analyzed the DL applications in BC histopathology, focusing on the impact of AI in diagnosis, prognosis and therapy of this major global women’s health concern [27,61]. In these reviews, B. Jiang and coworkers and Soliman A and coworkers analyzed the advancement of the performance of DL technology as a new potential tool in all steps of the clinical approach to BC, starting from diagnosis, grading, IHC typing, molecular characterization and prognosis to the prediction of metastasis risk and treatment response. The following fields were identified in these reviews in which AI models could be used to identify subtle features of tumor cells that are not appreciable at classical histopathology with a microscope at hand (Figure 2):

1. Histological grading. Histological grading is a process aimed at determining the aggressiveness and potential for spread of cancer cells based on their histological appearance. This grading is utilized, in clinical practice, to guide treatment decisions in BC patients [62]. In histopathology practice, mitotic activity, the number of mitotic figures in a given tumor area, is considered the most important grading component in BC [63]. More recently, immunoreactivity of phosphorylated Histone H3 (PHH3) has been introduced as an indicator of mitosis by revealing proliferating cancer cells in the M phase [64]. In recent years, a CNN model was proposed for detecting the mitotic index in H&E-stained WSI of BC after training on PHH3-immunostained sections [65]. This model showed the ability to define the BC mitotic index with similar accuracy to that of expert pathologists. The role of AI-driven models in BC grading and in the evaluation of the mitotic count has been confirmed in a recent review [61].
2. Histopathology of lymph node metastases. An algorithm, named smuLymphNet, has been developed to analyze axillary lymph node metastases, a finding associated with an increased risk of recurrence in BC patients [24]. Interestingly, this DL model was able to extract relevant information about cancer behavior, even in lymph nodes unaffected by cancer, through the quantification of germinal centers in triple-negative BC (TNBC) carriers. Lymph nodes with >2 germinal centers were associated with better prognosis and higher distant metastasis-free survival compared with patients whose cancer-affected lymph nodes showed fewer than 2 germinal centers. A study by Verghese et al. stresses the ability of AI models to effectively link some critical subtle features of axillary lymph nodes through their capacity to process WSI adeptly with BC patient prognosis [27].
3. Prognosis prediction based on histopathology. A DL model, named DeepGrade (DG), was proposed some years ago to evaluate the risk of recurrence in BC carriers based on H&E-stained WSI [49]. This model allowed the stratification of patients into two groups: DG1 and DG2. The latter were characterized by a higher risk of recurrence, suggesting that the AI-driven model could identify subtle histological features associated with a more aggressive BC subtype.
4. Tumor-infiltrating lymphocytes and prognosis. Tumor-infiltrating lymphocytes (TILs) are a very important tool for the evaluation of the immune response against BC cells [66]. In TNBC, TILs showed a correlation with improved prognosis and better response to immuno-oncology target agents [67]. Saltz J and coworkers showed that the spatial organization of TILs plays a key role and is associated with clinical outcome and prognosis [21]. Further studies based on the application of AI to assess the prognostic significance of TILs in luminal BC revealed that high stromal TILs and intra-tumoral TILs counts and their proximity to stromal and cancer cells were associated with poor clinical outcome, high tumor grade and lymph node metastasis. The spatial distribution of TILs and their relationship with cancer cells and with cells of the tumor microenvironment (TME) were evidenced by the AI model and were not assessed using the routine histological approach [68]. Another DL model confirmed that stromal TILs play a key role in predicting the response to neoadjuvant chemotherapy in BC patients [69]. In this study, the algorithm utilized appeared to be a useful tool for assessing prognosis and treatment response in both TNBC and HER2-positive BC carriers. All these data taken together suggest analytical and clinical validity of AI algorithms for the evaluation of TILs in BC [70].
5. Homologous recombination deficiency (HRD) prediction. HRD is a state where cells have difficulty repairing double-strand breaks. In BC, HRD is a significant factor in BRCA1 and BRCA2 mutations [71]. Recently, a DL model was proposed that is able to identify morphological patterns associated with HRD status in BC from H&E-stained WSI [72]. The model predicted HRD with high accuracy at an AUC of 0.86. The ability of AI-driven models to predict HRD from histology alone has been confirmed by more recent studies in which a new algorithm, named DeepHRD, predicted HRD without requiring molecular profiling in BC and ovarian cancer [73,74].
6. HR status prediction. HR status, including progesterone receptor and estrogen receptor expression, is an important factor for the stratification of BC patients into very high-, high- and low-risk subgroups, a key step for a proper treatment and prognosis [75]. DL models have shown their ability to enable HR status without the use of IHC, from base-level H&E-stained WSI [38,76]. The usefulness of AI in automated analysis of BC, including the prediction of HR status, has been confirmed by more recent studies [77].
7. Programmed Death Ligand-1 (PD-L1) expression. PD-L1 expression is an important biomarker for stratifying patients for PD-1/PD-L1 targeted immunotherapy. The first studies on the usefulness of AI models to assist PD-L1 scoring in BC were based on the analysis of BC sections immunostained for PD-L1 [78]. Further studies showed the ability of DL models to predict PD-L1 expression status from H&E-stained histopathology images in BC [79]. The proposed AI-assisted method was able to improve the ability and accuracy of pathologists in scoring PD-L1 expression [80,81].
8. HER2 status prediction. HER2 status represents an important prognostic and predictive marker in BC. The initial classification into two classes, HER2-positive and HER2-negative, has been successfully modified into a classification with three classes, including HER2 low status (score 1+ and 2+ without amplification) [82]. HER2 is a critical factor in BC treatment, and accurate differentiation of HER2 scores is crucial; therefore, AI has emerged as a promising tool for this challenging task. Tarantino and coworkers have developed an algorithm that differentiates between HER2-positive and HER2-negative BC [83]. Farahmand S and coworkers developed a DL model able to predict from H&E-stained WSI HER2 status and trastuzumab treatment response [26].

A recent meta-analysis of AI-driven models in classifying HER2 scores in BC demonstrated high accuracy in predicting survival benefits of trastuzumab–deruxtecan with a pooled sensitivity of 0.97 and specificity of 0.82 [84]. This meta-analysis confirmed that AI-driven models excel in distinguishing HER 2+ and HER 3+ scores, a critical point for therapeutic decisions.

9. Integration of histological data with multi-omics technologies. By combining histological, IHC, clinical, genomic, epigenomic, proteomic, transcriptomic and metabolomic data of a given patient, DL systems have been shown to provide relevant information regarding personalized treatment strategies for BC patients [85]. AI’s ability to integrate multi-omics might improve the development of precision oncology [86]. The topic of multimodal AI has been discussed in a recent study by Hanna MG and coworkers [87]. According to these authors, multimodal AI models may offer several advantages in oncology by integrating histopathologic, clinical, radiological and omics data.

## 8. Publicly Available BC WSI Datasets

A recent review of publicly available BC histopathology datasets useful for developing new algorithms in BC tissue identified 17 datasets [88]. In this article, the following most important datasets of BC WSI were reported: ACROBAT (4212 WSIs); ANHIR, BACH (30 WSIs); BCNB (1058 WSIs); BRACS (547 WSIs); Calelyon 16 (399 WSIs); Camelyon 17 (1399 WSIs); CPTAC-BRCA (642 WSIs): DRYAD, which includes The Cancer Genome Atlas (195 WSIs), the Cancer Institute of New Jersey (40 WSIs), the Case Western Reserve University (110 WSIs) and the Hospital of the university of Pensilvania (239 WSIs); GTEx-breast (894 WSIs); HER2-Warwick (86 WSIs); HEROHE (510 WSIs); IMPRESS (126 WSIs); Post-NAT-BRCA (96 WSIs); SLN-Breast (130 WSIs); TGCA-BRCA (3111 WSIs); TIGER, including WSIROIS (195 WSIs), WSIBULK (93 WSIs) and WSITILS (82 WSIs); and TUPAC16.

## 9. Methodological Challenges and Reproducibility in Computational Pathology

Although many studies report promising results, computational pathology research faces several methodological challenges that may limit the reproducibility and generalizability of AI models [89].

One important issue concerns dataset bias. Publicly available datasets may contain slides originating from a limited number of institutions, scanners, or staining protocols [90,91]. As a consequence, models trained on such datasets may inadvertently learn institution-specific or scanner-specific patterns rather than biologically meaningful features [92].

Another critical aspect is domain shift, which occurs when models trained on one dataset perform poorly on images acquired under different technical conditions [93]. Variability in staining intensity, tissue preparation, and scanner characteristics may significantly affect model performance [94].

Reproducibility can also be compromised by methodological issues:

- Overlap of patients between training and testing datasets;
- Tile-level data leakage during patch extraction;
- Lack of patient-level data splitting;
- Insufficient reporting of dataset composition and preprocessing procedures [95].

To improve transparency and reproducibility, reporting guidelines such as TRIPOD-AI and CONSORT-AI have been proposed for studies involving AI in healthcare [96,97]. Adoption of such standards may facilitate more rigorous evaluation and comparison of computational pathology models.

## 10. Regulatory Considerations and Clinical Implementation

The translation of AI-based tools from research settings into routine clinical practice requires compliance with regulatory frameworks governing software used for medical purposes [98]. In many jurisdictions, AI algorithms intended for diagnostic use are classified as software as a medical device and must undergo regulatory evaluation [99].

In the United States, approval of medical AI systems is regulated by the U.S. Food and Drug Administration [100]. Regulatory evaluation typically requires evidence of analytical validity, clinical performance, and safety [101]. Similar regulatory frameworks exist in Europe through medical device regulations governing AI-based software [102].

In clinical practice, most experts envisage AI systems functioning as decision-support tools rather than autonomous diagnostic systems [103].

In this scenario, AI algorithms could assist pathologists by highlighting suspicious regions, quantifying histological features, or providing decision-support outputs while the final diagnostic interpretation remains under human supervision [89].

Integration of AI systems into hospital environments also requires compatibility with digital pathology infrastructures, including slide scanners, laboratory information systems, and image management platforms [104].

In multidisciplinary oncology meetings, AI-derived quantitative features may eventually contribute to more data-driven discussions of tumor biology, prognosis, and treatment response [105].

However, prospective clinical trials and real-world implementation studies are still required to determine how AI-assisted pathology systems perform in routine diagnostic workflows [106].

## 11. Challenges in Computational BC Pathology

Despite the rapid progress of computational pathology research, several barriers continue to limit the translation of AI models into clinical practice [107]. First, many publicly available datasets remain relatively small and may not adequately represent the diversity of real-world pathology specimens. Dataset heterogeneity, including differences in staining procedures and scanner technologies, can influence algorithm performance. Second, external validation is still limited in many studies. Models developed using single-institution datasets may not generalize to other clinical settings [88]. Third, regulatory approval, quality assurance, and post-deployment monitoring represent essential steps before AI systems can be safely integrated into diagnostic workflows. A recent meta-analysis on the diagnostic accuracy of AI in different fields of digital pathology evidenced a lower diagnostic accuracy in the BC group compared to other cancer types [108]. In this study, the authors suggested that caution should be taken in the interpretation of any result of any AI-driven tool when considering its introduction in the real world of clinical practice [108]. These considerations highlight the importance of rigorous methodological design and transparent reporting in future computational pathology studies.

## 12. Future Directions in WSI Search Regarding BC

The development of AI is rapidly progressing across many areas of our daily life, and digital pathology is no exception [109].

The directions of pathologists and informatics involved in the development of new AI-driven models and in the application of publicly available ML and DL models are multiple. Here, we report some of the main directions in the actual research on digital pathology applied to BC.

- Development of novel multimodal models.
  - For pairing each WSI with other clinical, radiological and laboratory parameters.
  - For pairing each WSI with the patient’s clinical record.
  - For pairing each WSI with molecular tests.
  - For guiding diagnoses and clinical decision making.
  - To reach a system that can present a holistic view for pathologists, given a query WSI.
- Prepare large WSI repositories of BC.
  - Growing to millions of slides, the new datasets will allow DL systems to operate without pixel-level annotations.
  - The use of large datasets will favor the validation of novel algorithms.
  - The validation of new AI systems will favor their introduction in clinical practice and their acceptance in pathology departments.
- Development of fast and scalable search engines for multiplex transcriptomics and IHC data.

## 13. Conclusions

Pathologists are facing major changes in their daily practice, mainly due to the increasing workloads and lack of time to better analyze complex histopathological cases and perform a high-quality diagnosis, the basis for high-quality patient care. In this scenario, the application of AI to WSI within the pathology department workflow might significantly support pathologists in the provision of accurate and timely diagnoses [4]. Application of digital pathology, powered by WSI technology and by the novel algorithms developed for image analysis, has the potential to transform the landscape of BC research and diagnosis [110]. AI has emerged as a promising research tool in computational pathology, particularly for the analysis of BC histopathology images. DL algorithms have demonstrated the ability to extract quantitative information from digital slides and to assist in tasks such as tumor detection, grading, and biomarker prediction.

However, most AI models remain at the stage of experimental or research applications. Limitations related to dataset bias, lack of external validation, and regulatory considerations currently restrict their routine clinical implementation [111].

The need for the introduction of AI in pathology spans all fields of oncology, and accurate BC detection and prognosis might benefit from many algorithms developed for this purpose [85,107,112]. Furthermore, by applying microscopy and analyzing histopathological digitized images with machine learning or DL models, pathologists could identify the best algorithm to better classify and diagnose BC, including the most challenging subtypes, and to improve survival prediction [113,114]. Future progress in this field will likely depend on the availability of large multicenter datasets, standardized reporting practices, and prospective validation studies. Within these constraints, AI technologies may progressively contribute to decision support systems that enhance diagnostic workflows while maintaining the central role of the pathologist in clinical interpretation.

In conclusion, AI models have the power to significantly transform the activity of the pathology department, with major consequences for oncology departments, by changing the daily work of pathologists across all fields [53]. These changes will include a new AI-focused training of young pathologists; the introduction of scanners and WSI for histological diagnoses, including BC subtyping; the introduction of virtual staining and IHC staining; and the prediction of genomic changes from histological images, of treatment response from WSI analysis, and of OS based solely on cancer cell appearance and architecture at histology. Moreover, AI-driven models might help pathologists with assistance in the diagnosis of rare morphological findings, with primary site suggestion for metastases of unknown origin, and allow multimodal analyses, pairing pathological, clinical, laboratory, genomic and imaging data toward a holistic view based on WSI.

Thanks to these new skills, pathologists might be able to reinforce the linkages with oncologists, with a common goal: a better diagnosis for BC patients in shorter times, allowing them to receive early therapy based on novel therapeutic strategies, including precision oncology.

## Figures and Tables

**Table 1:** Representative studies applying AI methods to BC histopathology.

Study	Task	Dataset/Cohort	AI Approach	Key Findings
Bejnordi et al., 2017 [12]	Detection of lymph node metastases	Camelyon16 challenge dataset	CNNs	DL models achieved diagnostic performance comparable to pathologists in identifying lymph node metastases in WSI
Campanella et al., 2019 [20]	Tumor detection in histopathology slides	Multi-institutional WSI dataset	Weakly supervised DL	Demonstrated high sensitivity for cancer detection using slide-level annotations without exhaustive pixel-level labeling
Saltz et al., 2018 [21]	Spatial analysis of TILs	TCGA BC dataset	DL + spatial analysis	Spatial organization of immune cells was associated with patient survival and TME characteristics
Couture et al., 2018 [22]	Mitotic figure detection for tumor grading	Annotated histopathology images	CNNs-based detection models	Automated detection of mitotic figures demonstrated accuracy comparable to expert pathologists in grading tasks
Schmauch et al., 2020 [23]	Prediction of molecular alterations from histology	TCGA multi-cancer dataset	DL models	Demonstrated feasibility of predicting several genomic alterations directly from histological images
Verghese et al., 2023 [24]	Lymph node microenvironment analysis	BC lymph node dataset	DL WSI analysis	Germinal center quantification in lymph nodes correlated with prognosis in TNBC
Kather et al., 2020 [25]	Prediction of molecular biomarkers from histology	TCGA datasets	DL models	Demonstrated that histological patterns may correlate with molecular features across multiple cancers
Farahmand et al., 2022 [26]	HER2 status prediction	BC WSI dataset	DL classification	Demonstrated potential for predicting HER2 status from H&E slides with promising diagnostic performance
Jiang et al., 2023 [27]	Histopathological classification of BC	Public BC datasets	DL CNNs architectures	High classification accuracy for distinguishing malignant and benign breast tissue patterns

This table summarizes major tasks addressed in the literature, datasets used, methodological approaches, and principal findings. Reported studies illustrate the diversity of applications of DL in computational pathology, ranging from tumor detection to biomarker prediction and prognostic modeling. Abbreviations: AI, artificial intelligence; BC, breast cancer; CNNs, convolutional neural networks; DL, deep learning; H&E, hematoxylin and eosin; TILs, tumor-infiltrating lymphocytes; TNBC, triple-negative breast cancer; TME, tumor microenvironment; WSI, whole-slide imaging.

## Data Availability

All data analyzed during this study are included in this published article.

## Abbreviations

The following abbreviations are used in this manuscript:

AI	Artificial intelligence.
BC	Breast cancer.
CNNs	Convolutional neural networks.
DG	DeepGrade.
DL	Deep learning.
FDA	Food and Drug Administration.
HER2	Human epidermal growth factor receptor 2.
H&E	Hematoxylin and eosin.
HR	Hormone receptor.
HRD	Homologous recombination deficiency.
IHC	Immunohistochemistry.
ML	Machine learning.
OS	Overall survival.
PD-L1	Programmed Death Ligand-1.
PHH3	Phosphorylated Histone H3.
ROI	Regions of interest.
SISH	Self-supervised image search for histology.
TILs	Tumor infiltrating lymphocytes.
TNBC	Triple-negative breast cancer.
TME	Tumor microenvironment.
WSI	Whole-slide imaging.
